# GesID: 3D Gesture Authentication Based on Depth Camera and One-Class Classification

**DOI:** 10.3390/s18103265

**Published:** 2018-09-28

**Authors:** Xuan Wang, Jiro Tanaka

**Affiliations:** Graduate School of Information, Production and Systems, Waseda University, 2-7 Hibikino, Wakamatsu Ward, Kitakyushu, Fukuoka 808-0135, Japan

**Keywords:** gesture, authentication, depth camera, one-class classification, sparse autoencoder, neural network, incremental learning

## Abstract

Biometric authentication is popular in authentication systems, and gesture as a carrier of behavior characteristics has the advantages of being difficult to imitate and containing abundant information. This research aims to use three-dimensional (3D) depth information of gesture movement to perform authentication with less user effort. We propose an approach based on depth cameras, which satisfies three requirements: Can authenticate from a single, customized gesture; achieves high accuracy without an excessive number of gestures for training; and continues learning the gesture during use of the system. To satisfy these requirements respectively: We use a sparse autoencoder to memorize the single gesture; we employ data augmentation technology to solve the problem of insufficient data; and we use incremental learning technology for allowing the system to memorize the gesture incrementally over time. An experiment has been performed on different gestures in different user situations that demonstrates the accuracy of one-class classification (OCC), and proves the effectiveness and reliability of the approach. Gesture authentication based on 3D depth cameras could be achieved with reduced user effort.

## 1. Introduction

### 1.1. Background

**Figure 1:**
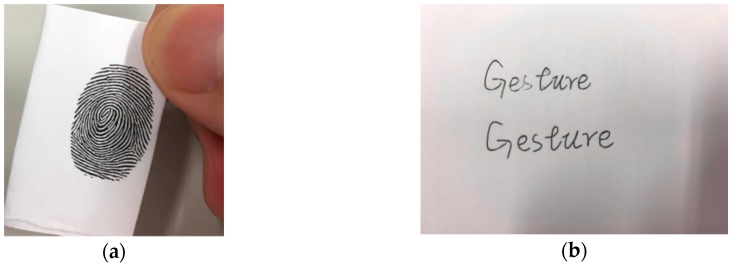
Different types of cheating on static physiological characteristics. (**a**) Is a fingerprint copy and (**b**) is imitation of handwriting.

In recent authentication research, biometric authentication has increasingly become a research hotspot. Biometric authentication can be divided into static physiological characteristics and dynamic behavioral characteristics. Static physiological characteristics refer to the technology of personal identification by using the physical characteristics that are inherent in the human body. Using static physiological characteristics, some applications such as Apple face ID have been widely used [1]. Although static physiological characteristics have made some progress, their weakness of being easily imitated still exists [2]. Fingerprint copying and imitating handwriting are examples of cheating, shown in Figure 1.

Because different users have different habits and different muscle memories [3], their dynamic behavior features are very difficult to be communicated by vision or experience, because these habits are not easily copied or imitated. As a type of dynamic behavior characteristic, gesture has three advantages [4]: Easy to express, large information content, and difficult to imitate. Therefore, it is suitable to be a carrier of authentication. Because the movement of a gesture can fully represent the dynamic behavior characteristic of a user [5], we aim to study the possible methods and the possibility of gesture-based biometric authentication.

**Figure 2:**
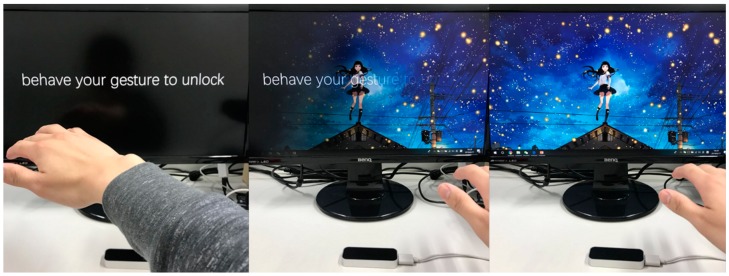
Unlock computer by gesture.

Consider the case shown in Figure 2, where a user wants to unlock their computer. They simply need to perform their own gesture “password” in front of his computer and the computer will be unlocked. The entire process is natural and smooth, and for this user, the gesture is also easily remembered. Of course, just as we need to set our text password first during registration, the system will also ask the user to register their gesture “password” and remember it during the registration stage.

**Figure 3:**
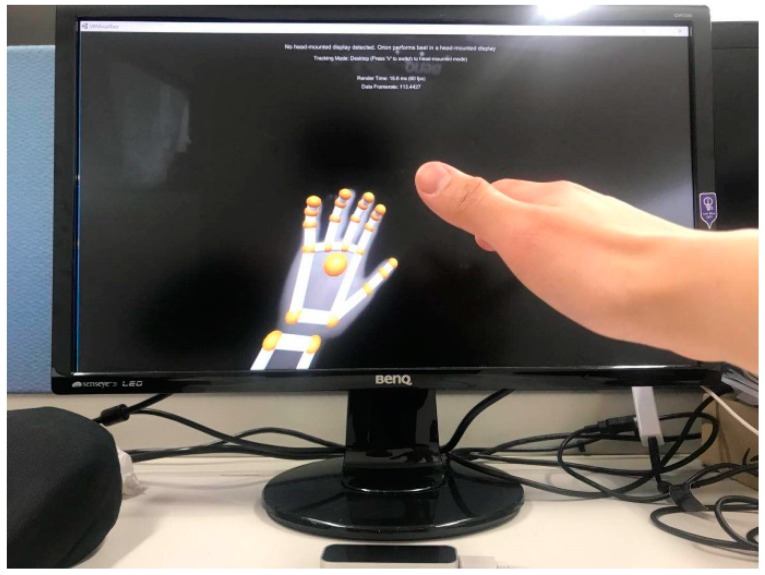
Working state of Leap Motion.

In this study, we use a depth camera called Leap Motion as the data input device (Figure 3), which is an infrared-based depth camera that can be used for tracking hands and fingers. To register a gesture, the user must gesture in front of Leap Motion several times. After the system learns the gesture model, the user simply performs the same gesture to unlock the system.

To make the system learn the user’s gesture more accurately, the system will continue to learn the gesture during use. This learning mechanism is very similar to human learning. As the knowledge is updated, the system needs to learn new knowledge and prior knowledge is also strengthened.

### 1.2. Goal and Approach

The fundamental goal of our research is to find a robust gesture authentication solution that requires little effort.

Researching dynamic characteristics like gestures as authentication methods requires significant effort in enlisting many users and collecting their gesture information from extensive participation. As this is not practical during realistic use, it is necessary to minimize this effort while ensuring high accuracy. The system should also satisfy these three requirements:

- Can authenticate from a single, customized gesture;
- Achieves high accuracy without an excessive number of gestures for training; and
- Continues learning the user’s gesture during use of the system.

To achieve these goals, we introduce our approach based on the several aspects. Firstly, three key aspects are introduced: One-class classification with autoencoder, data augmentation, and incremental learning.

To achieve highly accurate classification for a single user, gesture authentication is regarded as a “one-class classification” (OCC) [6] problem. Because of the particularity of the authentication, it is mostly conducted in a single-user scenario. Therefore, authentication can be considered as anomaly detection, and anomaly detection is an OCC problem. Unlike previous studies [7,8,9,10,11] in gesture authentication, a system based on OCC could be more accurately called “authentication” because it can directly identify the user without the need to rely on other user groups. Gesture authentication is mostly multi-class classification in the previous studies, which means the system can only distinguish which user is the “authorized user,” but it cannot directly identify the user without other information. Also, the requirement of multi-class classification can be realized when multiple OCC systems are assembled together. An autoencoder is used as our OCC model, and this autoencoder is a five-layer neural network. It compresses the input data into a feature vector and then decompresses the feature vector into its original size. A mean squared error loss function will be used to evaluate the differences between input and output as a labeling process. Therefore, the autoencoder learns the positive gesture in the training stage; in the test stage, it will map the high-dimensional gesture data to a one-dimensional mean square error interval. For identifying true and false classes, we propose a boundary making strategy to judge if the test data is in a positive interval or a negative interval.

Data augmentation is employed to overcome the difficulty that deep learning requires a large amount of data to ensure the accuracy of the results. Data augmentation [12] is a very effective solution in the case of insufficient data, as the supplemental data is added by performing specific operations on the data.

Further, incremental learning technology is used to allow the system to continue learning the gesture while the user is operating the system. Incremental learning [13] is a human-like learning mechanism of machine learning, as it will acquire the new knowledge without forgetting prior knowledge. Incremental learning will increase the accuracy of the system by re-learning the model during system use.

To capture three-dimensional (3D) dynamic gesture movement, Leap Motion is employed as our data input device. Because depth cameras are gradually gaining popularity, their use will increase in our daily lives. This will render depth-camera-based authentication highly effective. 

To render the Leap Motion more robust for the system, we use a series of data preprocessing, including filtering and data normalization, to achieve a more effective and robust algorithm. These methods are mature in many current situations. Because Leap Motion will produce noise and the data position is not centered, we significantly enhance the processing to improve the result. It is believed these types of processes are very important for study tasks and for business.

### 1.3. Related Work

There are a number of studies on different input devices and types of gesture authentication systems. Clark et al. [7] surveyed gesture-based authentication attributes and methods. Their conclusions conveyed promising prospects for gesture authentication. Chong et al. [8] used mobile phone sensors to create templates by capturing different parameters of gestures. By performing several gestures, they established different templates for different users. Further, experiments were performed to verify the accuracy of their approach.

Kinect is a type of depth camera, and Shukran et al. [10] proposed a Kinect-based gesture authentication system. They employed the Baum-Welch algorithm to classify the different gestures. 

Liu et al. [14] also proposed a system using a dynamic time warping algorithm to process 3D accelerator data. Further, a series of experiments were performed to evaluate the performance of their algorithm. Sae-Bae et al. [9] proposed an approach using multitouch gesture-based authentication using feature computation and a distance function strategy. Aumi et al. [15] proposed a high-performance system for gesture authentication using a distance-based template-matching algorithm. They also endeavored to make their system easy to use.

Kamaishi et al. [16] used Leap Motion as the input device and presented several cases about how gesture authentication could be used realistically. They used a series of comparisons to determine if this gesture belonged to the user. Zhao et al. [17] also used Leap Motion with a hidden Markov model as the classification method for multi-class classification. Aman Chahar et al. [11] proposed a system also based on Leap Motion, using a conditional mutual information maximization algorithm to select the optimal feature set. Match-score fusion was performed to reconcile information from multiple classifiers. They also compared different methods and evaluated their approach.

In summary, there are several studies on gesture authentication. The device, algorithm, and user cases have many differences but also share some common properties. The common properties mainly relate to the accuracies of the algorithms, because authentication requires very high accuracy to achieve reliable security.

## 2. Method

**Figure 4:**
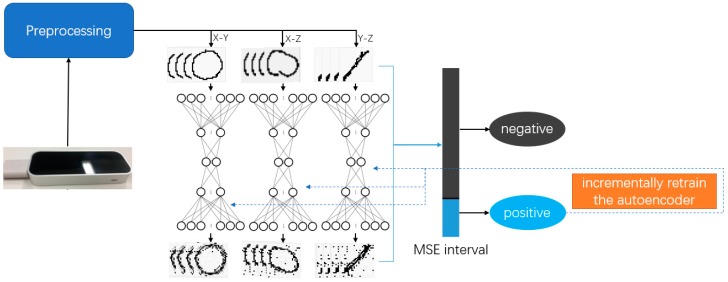
Algorithm flow chart.

In this section, we introduce our approach, describing each step of the algorithm. Figure 4 shows the overall structure of the algorithm. First, it will collect the data from Leap Motion, and then median and Gaussian filters will be applied to smooth the input data. Subsequently, 3D data will be mapped to three two-dimensional (2D) images in planes of X-Y, X-Z, and Y-Z. Three sparse autoencoders (SAEs) will classify these input data as the gesture of the legal user or not, which is an OCC process. If the gesture passes the authentication, this batch of data will be re-used for incremental learning to generate improved results in further use. The emphasis is on SAE, the boundary making strategy, and incremental learning.

### 2.1. Data Collection

**Figure 5:**
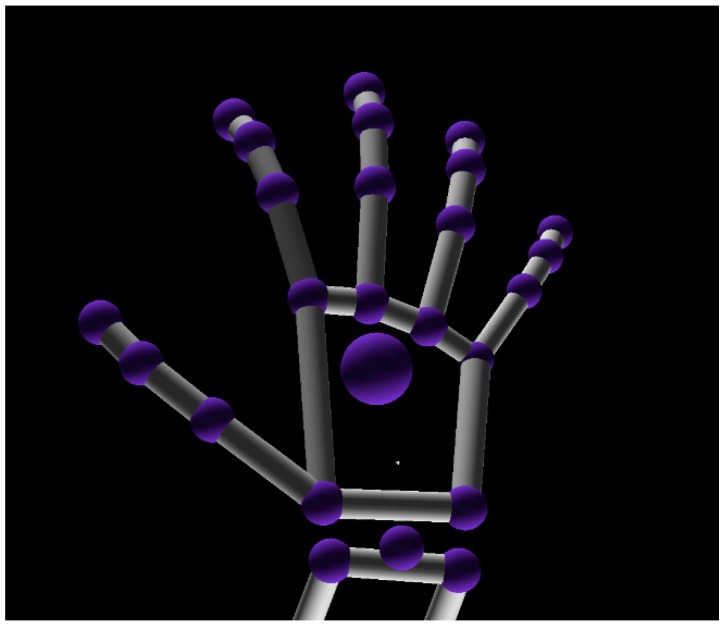
Hand model in Leap Motion.

In our proposed system, Leap Motion [18] is selected as the data source to collect raw gesture data. Figure 5 shows the image captured from its viewer. Leap Motion is a very small device that has different types of infrared-based depth cameras inside, specially designed for tracking and detecting human hand movement. The data from Leap Motion contains abundant information (such as location and velocity) in a virtual coordinate system.

We have considered several input devices for gesture information collection, such as the devices used in previous research [7,8,9,10,11]. There are two primary reasons for choosing this device. First, the depth camera is current, and has a promising future in many fields. Second, this device is suitable for tracking hand movement and includes significant performance optimization.

For deciding what type of data to obtain from gestures for further processing, another study was investigated [5]. In the generalized concept of gesture, there are several other types of gesture besides hand gesture, such as arm gesture. It was found that the moving state of the hand is a key component of gesture; therefore, we decided to capture fingertip data for five fingers, including position and velocity information.

A buffer is used to save information of every frame from Leap Motion. According to the moving state (move or stay), the system will decide whether a gesture is behaving or behaved completely. The moving state depends on the square variance of last several frames, if the square variance is under a threshold, the state will be judged as stay, otherwise will be judged as move. If the gesture is behaved completely, the raw data in buffer will be released and will be ready for further processing.

### 2.2. Preprocess

#### 2.2.1. Filter

As filtering technology is a very mature technology, we introduce the filter very briefly. In early tests, it was found that there were two problems caused by the device in the data transmission process: (1) The data flow was not stable, and isolated points (salt and pepper noise) would be generated from time to time; (2) The data was not smooth and produced fluctuations. As gesture authentication does not require very detailed data points, we chose to use a fuzzy filter to process the data. Median and Gaussian filters were used to solve these two problems, respectively.

The median filter is used first. The dataset from the depth camera is a 3D point cloud dataset, therefore a 3D median filter with a window size of five will be used, and the filtering process is defined by the following formula:

(1) g[x, y, z]=med{f[x, y, z], (x, y, z)∈W},

where W is the set of front and rear five data points of the current data as the sliding window; g[x, y, z] represents the 3D coordinates of a filtered point; and f[x, y, z] represents the data before filtering. Outliers will be filtered out, meaning there will be no mutational point in the data, which would have an obvious effect on the model’s classification result.

Because median filtering is non-linear, the unsmooth characteristics of the data still exist. Here we use the Gaussian filter with filter kernel size of 5 × 5 × 5, σ = 0.8. The values of each point in the kernel will be determined by the following Gaussian distribution:

(2) $$G\left(x,y,z\right)=\frac{1}{2{\pi \sigma }^{2}}e^{-\frac{x^{2}+y^{2}+z^{2}}{2\sigma^{2}}},$$

where σ is the standard deviation of the entire data. This formula is the principle of Gaussian kernel generation. After obtaining the Gaussian kernel, we perform a 3D convolution operation with the data.

#### 2.2.2. Normalization and 3D Mapping

As the dataset we obtain is in three dimensions (i.e., one gesture is characterized by three features), each gesture will be measured by establishing a 3D coordinate system. In addition, the position relative to the device is usually different for each gesture occurrence, and the coordinates of movement track are also different. If these different coordinates are interpreted as in the same coordinate system, errors will be produced during the subsequent learning process. Even in the current study, neural networks have been size-insensitive. The size-sensitive data input will have better fitting results (i.e., more accurate than that without data normalization). Accordingly, the data is normalized after filtering. Its purpose is to eliminate irrelevant differences within each gesture ensuring the model can learn the key data. Further, the importance of normalization has been discussed by previous research [19].

**Figure 6:**
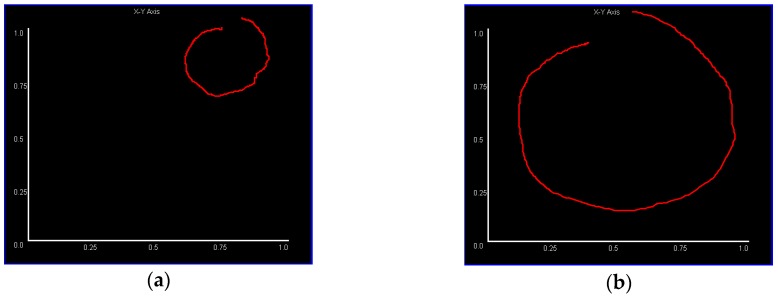
This figure shows similar gesture (“circle” gesture mapped in X-Y plane) but in a different position relative to device, where the red line is the mapping of a 3D gesture. (**a**) This gesture is farther and more biased from device. (**b**) This gesture is appropriately behaved on the center and top of the device.

If the position and size of every gesture are different, it will “refuse” our neural network, increasing the false judgement rate. We need to transform the data from an absolute coordinate system into a relative coordinate system, besides, we also need to rescale the data into a same standard of size. For example, Figure 6 is made for comparing if same gesture behaving in different position what will happen. 

Figure 6 shows the difference between similar gestures (for simplicity, in this figure gesture movement is mapped only to the X-Y plane) in different relative positions to the device. If there is no normalization operation, a “congenital” input error will exist, and it is very difficult for a classifier to judge if the two gestures are similar.

Here, our normalization method is to scale the gesture data in proportion to a small specific interval, remove the unit limit of the data, and convert it into a pure value. To make the next step of classification more accurate and easier to perform, we also map the original 3D data into three sets of 2D data. We use a “window scanner” to scan this 3D movement and produce the resulting 2D data, which can also be regarded as an image. To make the classification step more accurate and easier to perform, we define the size of the mapping result as 28 × 28 and every point of this result is 0 or 1, thus 784 data points will be used for the next classification.

Our algorithm for normalization is performed by the following steps:

(1) Generate 676 (=26 × 26) 2D scanning windows. The start location of a window can be described as follows (here we take the mapping of the X-Y plane as an example; X-Z and Y-Z planes are handled similarly):
  (3) $$w_{i,j}=\left[26i\left(x_{\max}-x_{\min}\right),\;26j\left(y_{\max}-y_{\min}\right)\right],$$
  where $w_{i,j}$ is the start location of scanning window, $x_{\max}$ is the max value in the X dimension of the gesture, and $x_{\min}$, $y_{\max}$, and $y_{\min}$ are similar. The reason we use the window size 26 × 26 instead of 28 × 28 is to prepare for the next data augmentation. Please refer to the data augmentation below for details.
(2) For each window, scan the specific plane (e.g., X-Y plane’s scanning means only X and Y dimension’s data will be used) of gesture. If there exists at least one data point, the mapped result will be 1, otherwise it will be 0, represented as follows:
  (4) $$r_{i,j}=\{\begin{array}{l} 0,\;\;\;\mathrm{there}\;\mathrm{exists}\;\mathrm{positive}\;\mathrm{value}\;\mathrm{in}\;w_{i,j}\\ 1,\;\;\;\mathrm{there}\;\mathrm{are}\;\mathrm{all}\;0\;\mathrm{in}\;w_{i,j} \end{array},$$
  where $r_{i,j}$ means the point in the result matrix;
(3) Generate four edges of the result, adding a row (or column) of all 0 values to the top, bottom, left, and right.
(4) Repeat steps (1) to (3) until the three planes are all scanned.

**Figure 7:**
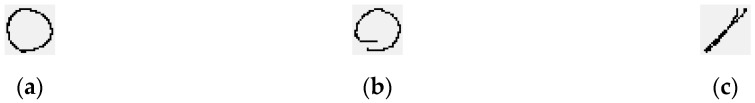
The images resulting from data normalization and 3D mapping. (**a**–**c**) Are the mappings of X-Y, X-Z, and Y-Z planes respectively. Because the “circle” gesture is 3D, the images in the X-Z and Y-Z planes may not look like circles.

To illustrate the results of this process more intuitively, Figure 7 shows the example of a “circle” gesture in three different planes after processing (we generate images to observe the result, but it looks very small because the size is 28 × 28).

**Figure 8:**
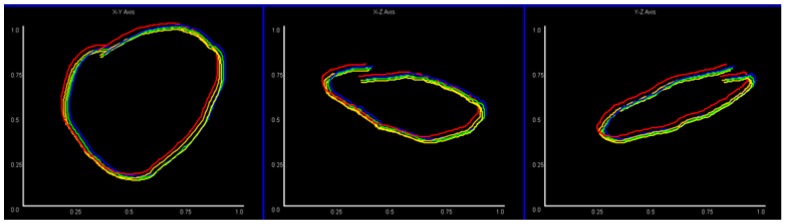
Real-time interface of the gesture movement. The data has been normalized and these 5 colors represent five finger movements. The X-Y, X-Z, and Y-Z plane’s gesture mapping are shown from left to right.

For convenience, we also developed an interface to facilitate observation and operation. Figure 8 illustrates the interface for three planes when users are gesturing. Figure 7 shows the resulting image file, and Figure 8 is the real-time interface during operation (the gesture in Figure 8 is different from Figure 7).

#### 2.2.3. Data Augmentation

In the consideration of practical algorithm design, we find that requiring users to perform a lot of times (e.g., 100 times) of gestures is unrealistic. However, deep learning usually needs considerable data to train the network. Based on previous research [12], data augmentation is used to expand the dataset. Another advantage of data augmentation is that it can reduce data overfitting. Through the transformation of the training pictures, the network’s generalization ability improves, and it is more applicable to the scenario.

To maintain the size and shape, we use shift, flip, and rotation as data augmentation operations, instead of choosing operations such as zoom in and out. Under these operations, each original dataset could become 160 datasets, which considerably expands the scale of the dataset.

**Figure 9:**
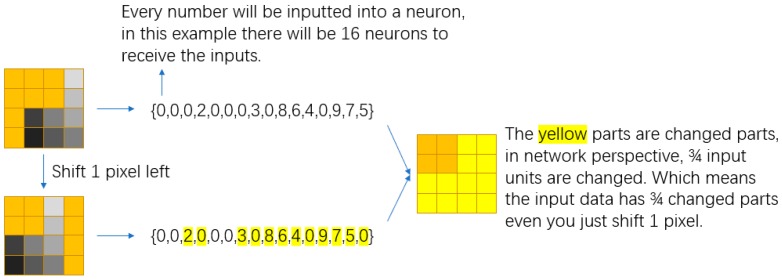
The input comparison of original data and shift 1 pixel left.

The reason is, the neural network will receive a totally different input even if the data is just shifted for only one pixel, although they look almost the same in morphology. For example, A is augmented as A’, they look almost the same, but most of the neuron will receive a totally different input. In network perspective, A and A’ is totally two data, and the network needs to dig the pattern between A and A’. Furthermore, there will exist a huge amount of A’, which allows the network learning to be more simple with less input. Figure 9 is the example to intuitively describe this.

### 2.3. One-Class Classification with Sparse Autoencoder

When the classification problem is mentioned, in most cases there are two or more classes of data to be recognized. Data and their labels are input into a classifier model for learning, and when finished the model can be used for classifying new data.

However, there could be a problem if in the training process there are only positive data and no other data as a contrast. In other words, there is only one label assigned. The key problem is that when the trained model is in use, negative data will arrive, and the model must classify negative data correctly. However, there were no samples of negative data in the training process, and negative data can vary. This type of problem is one-class classification (OCC), as mentioned in the introduction. The problem is also referred to as anomaly or outlier detection. The solution is to draw the boundaries of positive data. Any data falling within the boundary will be considered as positive data, and all data outside the boundary will be considered as negative data.

In the case of gesture authentication, we assume that all data are positive data during the training process, and there would be negative data during use, so it is a typical OCC problem. Classifying user data and non-user data in this problem is also an important issue.

From a survey [20] of previous research, the current solutions are mainly divided into one-class support vector machine (OSVM) and non-OSVM (such as hidden Markov model and nearest neighbor). As these methods play a very important role in the development of one-class classification, a detailed comparison is given in the Experiments and Results section. Our method uses an SAE as the classifier, and the boundary is constructed by a specific strategy.

#### 2.3.1. Structure of Proposed Sparse Autoencoder

An autoencoder [21] is an unsupervised feed-forward neural network. It is composed of an encoder and a decoder. The input data are compressed in the hidden layer and then decompressed to its original size. Because the compression is lossy, the autoencoder can approximately copy the input, and the useful features of the data will be learned. Autoencoders are data related. Further, if an autoencoder has learned a class of features, then compression of this class will have a good result, but the compression of other classes will be very poor. The encoding process can be represented as follows:

(5) $$h=f_{\mathrm{enc}}\left(Wx+b\right),$$

where h∈ℝ is the hidden feature vector of the autoencoder, x∈ℝ is the input data matrix, W is a weight matrix, and b is the bias vector. The autoencoder will also decode this feature vector:

(6) $$x^{\prime }=f_{\mathrm{dec}}\left(W^{\prime }h+b^{\prime }\right),$$

where $x^{\prime }$ is final output of the autoencoder, and $W^{\prime }$ and $b^{\prime }$ are the parameters in the decoding process. Terms $x^{\prime }$ and x are similar and the autoencoder minimizes the difference between them.

The learning process of an autoencoder is to compare the input and output, and to train the autoencoder according to the loss calculated by the random gradient training algorithm. Our loss function will be:

(7) $$L_{\mathrm{MSE}}\left(x,x^{\prime }\right)=\frac{1}{n}\sum_{i=1}^{n}\|x-x^{\prime }{\|}^{2},$$

where $L_{\mathrm{MSE}}$ is the loss function that is used to penalize the difference between input and output with n predictions. There are several functions that can be used as a loss function. We selected mean squared error (MSE) as the loss function. Because our input and output are 2D data that can be regarded as images, the MSE score is an effective measurement.

A sparsity penalty is used to make the feature vector better express its learning features, and to make the output more mixed for non-standard input. In other words, the sparsity penalty can make the autoencoder have a better specific expression for input already learned. The autoencoder with this sparsity penalty is an SAE [22]. If we want the average activation of neurons to be small, we must add a sparsity penalty to the final loss calculation. If $\rho_{j}$ is the activation degree of neuron in the hidden feature vector layer, then the equation is:

(8) $$\rho_{j}=\frac{1}{n}\sum_{i=1}^{n}\left[a_{j}\left(x_{i}\right)\right],$$

where $a_{j}\left(x_{i}\right)$ represents the activation degree of neuron j of input data matrix $x_{i}$. When calculating the sparsity, SAE uses Kullback-Leibler Divergence (KL Divergence) as a sparsity penalty. Assuming that ρ is the required average activation degree, then the final cost function is as follows:

(9) $$C=L_{\mathrm{MSE}}\left(x,x^{\prime }\right)+\beta \sum_{j=1}^{m}\mathrm{KL}\left(\rho ∥\rho_{j}\right),$$

and

(10) $$\mathrm{KL}\left(\rho ∥\rho_{j}\right)=\rho \log\frac{\rho }{\rho_{j}}+\left(1-\rho \right)\log\frac{1-\rho }{1-\rho_{j}},$$

where β is the weight parameter of the sparsity penalty, and m is the neuron number of the hidden feature vector layer. From the loss, we can train the autoencoder through the stochastic gradient descent method.

**Figure 10:**
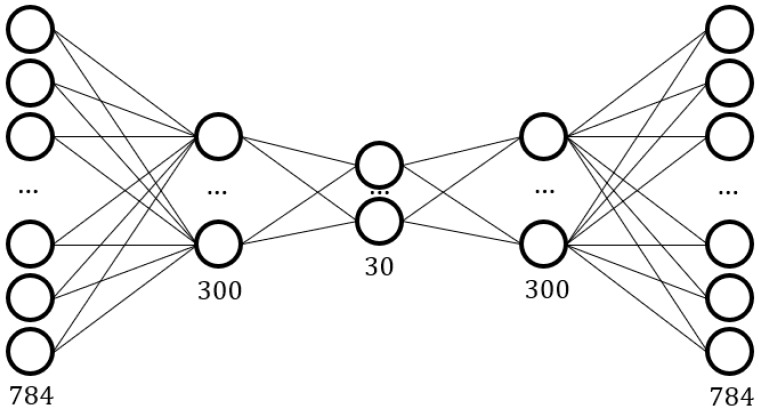
Network structure of the Autoencoder.

Figure 10 displays the structure of our autoencoder. This network structure was chosen to balance the best match reduction degree and specificity matching based on the results of many experiments.

#### 2.3.2. Network Parameters and Training Process

**Figure 11:**
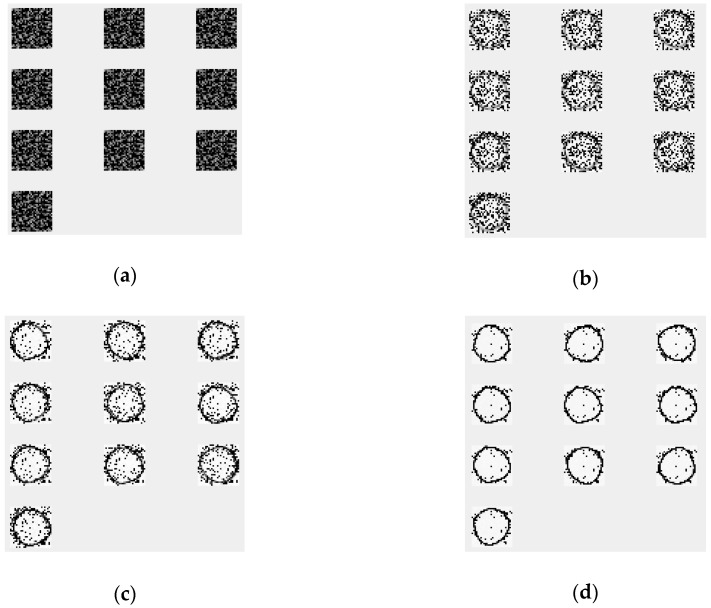
Selected training stages from 6000 iterations for the input gesture “circle”. This figure only shows plane X-Y’s training. X-Z’s and Y-Z’s training look different because there are three autoencoders training them separately. (**a**) Is in the initialization stage (iteration = 1); (**b**) is iteration = 500; (**c**) is iteration = 2000; and (**d**) is the final stage (iteration = 6000).

Neural networks require pre-configuration (hyperparameters) to achieve the best relative accuracy. For the selection of these hyperparameters, we conducted several experiments with different settings. As a result, rectified linear unit (ReLU) is set as the activation function, and we perform a 30-epoch training procedure to prevent overfit. For intuitive impression, we plot the training results of the output in every iteration, as shown in Figure 11.

Figure 11 shows the training process of different iterations in gesture “circle”. As the number of iterations increases, the output results of the autoencoder better fit the input. The fitting does not have zero error; there are some errors in the reduction of the input. The establishment of this model provides a standard reductive output for specific inputs. As mentioned previously, the output and input can be compared, and the standard of difference is the MSE score. A lower MSE score indicates that input and output are the more similar in shape, while a higher MSE score indicates greater difference.

**Figure 12:**
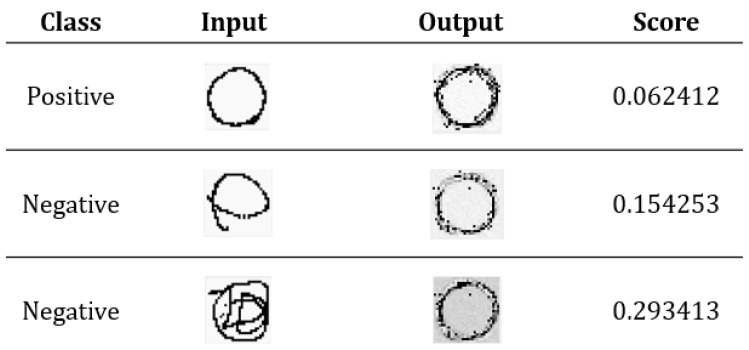
Different scores for different inputs.

Figure 12 provides the scores for different tests. It is remarkable that the input in Figure 12 is the test dataset, not the training dataset. The output is produced by a trained autoencoder (as shown in Figure 11). From that figure, the higher the similarity between the test and training dataset, the higher the reduction degree (i.e., the output will be more like the training dataset). If the input of the test dataset does not match the input of the previous training, the output will become very chaotic.

### 2.4. Boundary Making Strategy

Thus far, our algorithm has been able to map the input of gestures to a one-dimensional (1D) linear interval, but how to classify such a 1D linear space remains a problem. Nathalie Japkowicz et al. [21] first proposed a threshold determination approach that relaxes the last training score by 25% higher than the score. Larry Manevitz et al. [23] optimized that idea but tightened the training result and proposed another strategy using the score at the 90th percentile, because in that stage the training score usually is not the best score. In other words, the score at the 90th percentile is a little higher than the last, which can be regarded as the boundary. These ideas are very enlightening, and based on the study of this research we propose our boundary making strategy.

During training of the network, we find that the training curve has fluctuating characteristics. The degree of fluctuation is different for different gestures. The key to our border making strategy is to choose the boundary loose or tight according to the fluctuation of the data. The boundary should have higher capacity when the fluctuation of training data is relatively large; while the boundary will be more contracted when the training data is relatively stable. In addition, the MSE score is gradually converging during autoencoder training. In other words, there will be a gradual optimizing trend, therefore, the score of the last part of the training will produce better results.

**Figure 13:**
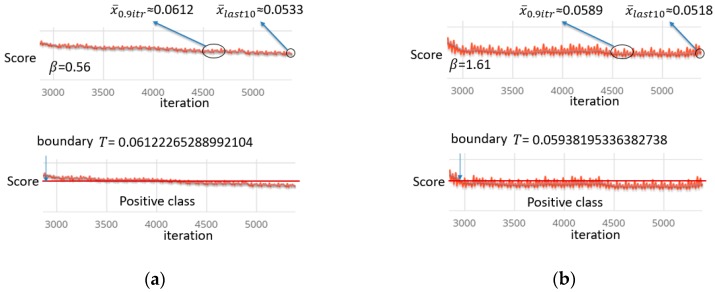
Example fluctuation and boundary situations. The curve is the intercepted segment of last part of training. (**a**) Is a relative stable situation while (**b**) is relatively unstable. T is the boundary shown by a red line.

In summary, we propose the following strategy for boundary making:

(11) $$T=\left(1+\beta^{2}\right)\frac{T_{0.9\mathrm{itr}}\cdot T_{\mathrm{last}10}}{\left(\beta^{2}\cdot T_{0.9\mathrm{itr}}\right)+T_{\mathrm{last}10}},$$

and

(12) $$\beta =\sum_{i=0.6\mathrm{itr}}^{n}\left|x_{i}-x_{i-1}\right|,$$

(13) $$T_{0.9\mathrm{itr}}={\bar{x}}_{0.9\mathrm{itr}},{\;T}_{\mathrm{last}10}={s\bar{x}}_{\mathrm{last}10},$$

where T is the threshold, β is a proportion and represents the fluctuation degree, s is relaxing parameter (set as 1.15, which means it is relaxed by 15%), ${\bar{x}}_{\mathrm{last}10}$ is the last 10 iterations’ mean MSE value, and ${\bar{x}}_{0.9\mathrm{itr}}$ is the mean MSE value of 50 iterations at the 90th percentile. This strategy balances $T_{0.9\mathrm{itr}}$ and $T_{\mathrm{last}10}$ by the proportion parameter. We performed 50 experiments and found that the value is between 0.35 and 1.73, depending on the different fluctuation situations. Figure 13 shows different fluctuation and boundary situations using our boundary making strategy.

**Figure 14:**
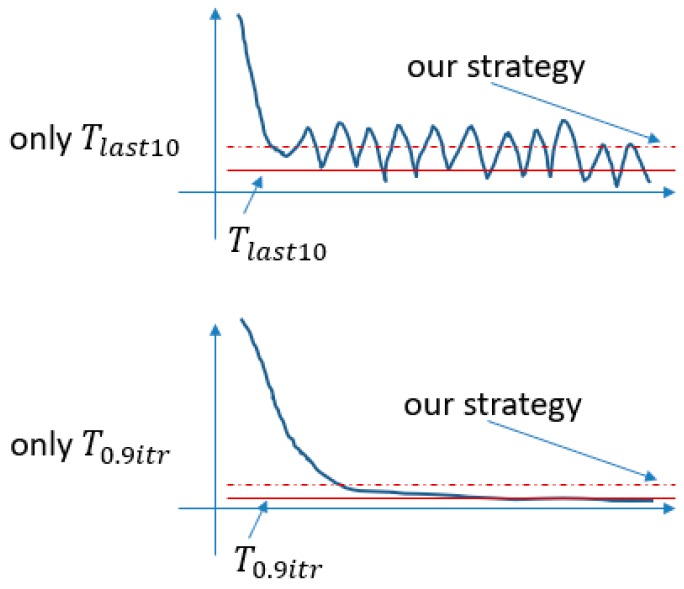
Schematic diagrams to explain the problems when only one threshold is used.

The main reason for this strategy is to avoid the problem that happens when any of the two strategies is used alone. If only the final results ($T_{\mathrm{last}10}$) are used, the final segment shrinks to the lowest point, but only the local lowest point, and the boundary setting does not have good classification characteristics. If only the 90th percentile’s mean scores ($T_{0.9\mathrm{itr}}$) are used, the final segment is very smooth and hardly experiences any optimization. The score located at the 90th percentile is almost the same as the last score, which also does not have good classification characteristics. We drew two schematic diagrams to represent the two problems, respectively, in Figure 14.

### 2.5. Incremental Learning

If a learning system has the ability to gradually learn new knowledge from a new batch of data, and to preserve knowledge that has already been learned, this system has the ability of incremental learning. This learning mode is much like a human learning mode, because like a human who learns and receives knowledge every day, most of the prior knowledge will not be forgotten.

Our approach will gradually learn the new batch while the system is being used. Assuming the user performs his gesture and passes authentication, this batch of gestures will be re-trained to fit the autoencoder with a higher learning rate and less number of epochs, then generate results in the direction of gradient decent. The incremental learning will not allocate a special session for user to ask user to behave his gesture instead this process will happen at every time the user authenticate successfully. This will let our autoencoder memorize the gesture incrementally imperceptibly over time.

There are two main reasons for using incremental learning. One reason is our user-friendly consideration that it is not practical for a user to perform several gestures during the training stage. Using incremental learning will solve this problem, because it prevents the need for several gestures. Another reason is that incremental learning will increase the accuracy during use, because the autoencoder gradually learns the user habits, and from the unique data of others that will largely reduce the false rejection rate of the system.

An evaluation of incremental learning is provided in the following section.

## 3. Experiments and Results

**Figure 15:**
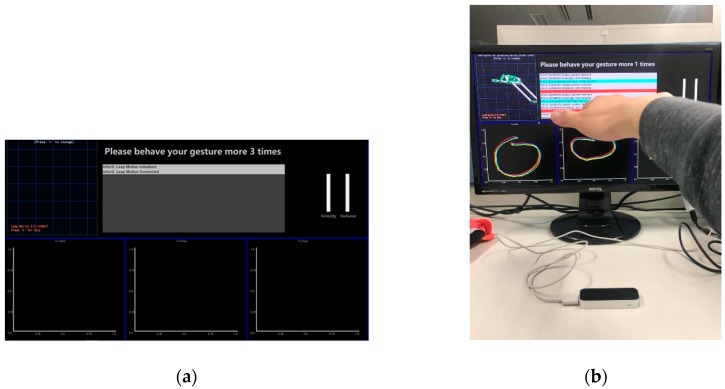
The gesture authentication system we developed for experiments. (**a**) Is the gesture interface. On the top are the real-time 3D gesture, console, bars of velocity, and variance from left to right. The X-Y, X-Z, and Y-Z gesture mappings are on the bottom. (**b**) Illustrates the use of the interface.

The experiment environment, shown in Figure 15, is based on our system we have developed to conduct gesture authentication as described in Section 2.

**Figure 16:**
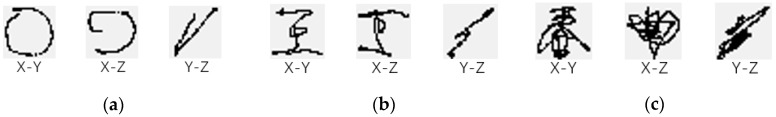
The three planes mapping of three gestures. (**a**) Is the simple gesture, (**b**) is the normal gesture, and (**c**) is the complex gesture.

To prove the performance of authentication and our approaches are work or not, the experiments aim to verify the accuracy of user gestures and the accuracy when false gestures are performed. We focused on the performance of different situation of every single user, and we will make a series of analysis to test if our technologies are work and if these technologies have bad influence on the performance. As there was no public database meeting our requirements, we constructed the experiment dataset. Because OCC technology enables our system could recognize user’s gesture without others’ false sample, we would not construct a big user set. Also, the comparison of other works in number of user set will be given in Section 3.5. We enlisted six users aged 22 to 25 who had no experience with this type of system but they were taught how to use our system as our research objects, the data of every time that they interact with our system will be saved, and we will analyze the performance through their using process:

1. At registering stage, two users will be asked to perform a simple gesture, two users will be asked to perform a normal complexity gesture, and two users will be asked to perform a complicated gesture. Figure 16 shows the sample of simple, normal, and complicated gestures (every user will gesture three times); Then users will prepare 20 times’ positive gestures as true class for further analysis.
2. Users are then asked to authenticate their own gesture 20 times, the autoencoder model will be saved after every time finished. (Incremental learning is used in every time of authenticating successfully, and the autoencoder will learn the new knowledge.);
3. Each user’s gesture will be tested by the other five users. Each user will test four times, totaling 4 × 5 = 20 times for each user. This is an attack experiment.

For performance evaluation, we used the false acceptance rate (FAR) and the false rejection rate (FRR), and for an intuitive and detailed view, we used the receiver operating characteristic (ROC) curve for further explanation. 

**Figure 17:**
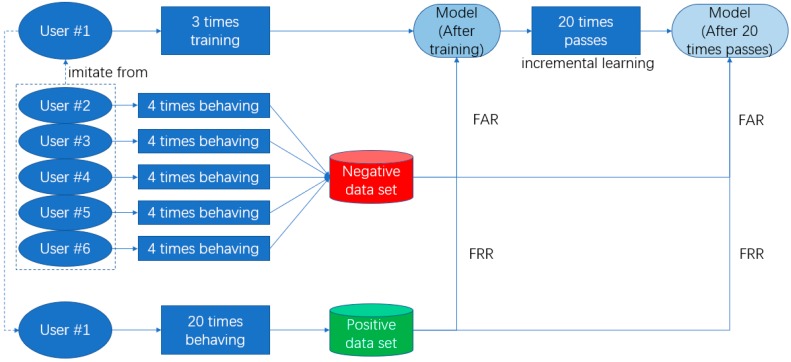
User #1’s perspective of experiments.

If the model classifies the gesture as a positive gesture and the gesture is a positive gesture, the classification is a true positive (TP) classification. Further, if the model classifies the gesture as negative and the gesture is negative, it will be regarded as true negative (TN) classification. False positive (FP) is the improper prediction that a negative gesture is positive and false negative (FN) is predicting a positive gesture as negative. FAR and FRR are defined as follows: FAR = FP/(FP + TN) and FRR = FN/(TP + FN). Figure 17 is made for an intuitive view.

The receiver operating characteristic (ROC) curve is a comprehensive indicator reflecting continuous variables of sensitivity and specificity. Usually this curve is used to evaluate the performance of a classifier, where the horizontal axis is the false positive rate (FPR) and the vertical axis is the true positive rate (TPR). FPR and TPR are defined as follows: FPR = FAR = FP/(FP + TN) and TPR = TP/TP + FN.

### 3.1. Analysis on False Rate

In this section, the basic accuracy for these six users is analyzed.

Our experimental results are shown in Table 1, including the result after training (just registered, and 3 gestures) and after 20 passes (the model has been incrementally retrained 20 times).

Users 1 and 2 are performing simple gestures, users 3 and 4 are normal, and users 5 and 6 are performing complicated gestures. Results show that the score after 20 passes is better than just after training (The detailed test of incremental learning will be shown in Section 3.4).

### 3.2. Comparison of Simple, Normal, and Complicated Gesture

**Figure 18:**
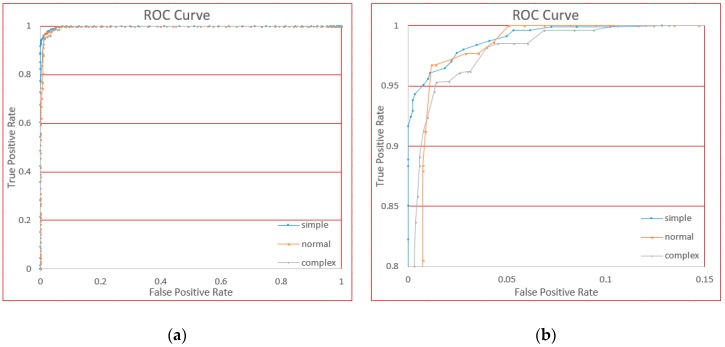
The receiver operating characteristic (ROC) curves for three gestures. (**a**) Is the global view and (**b**) is the local view for clarity.

In this section, we will demonstrate the performance for the different complexities of gesture: Simple, normal, and complicated. We used the original data from six users’ gestures, and by manually adjusting the threshold value, we generated ROC curves, as shown in Figure 18.

From Figure 18, we see that the curve fits almost to the upper left corner. In an ROC curve, the closer the curve is to the upper left corner, the better the classifier. 

For three different gestures, we can see the TPR value is higher than 95% where FPR is approximately 1% from the curve. We can see that regardless of the complexity of the gestures, our approach has very good performance. Further, the FPR performance of the simple gesture is very good; on normal and complex gestures, the performance is acceptable.

### 3.3. Analysis on Data Augmentation

In this section, the data augmentation effect on performance of our approach is analyzed.

The original “circle” gesture used data augmentation by gesturing three times. The original user participated in two additional scenarios: 15 repetitions and using just a shift operation; and 60 repetitions and without any data augmentation operation.

Table 2 shows the FAR and FRR of these three groups. We can see that data augmentation has little influence on the FAR and FRR. This is because our boundary strategy establishes the threshold according to the different situation of every gesture.

**Figure 19:**
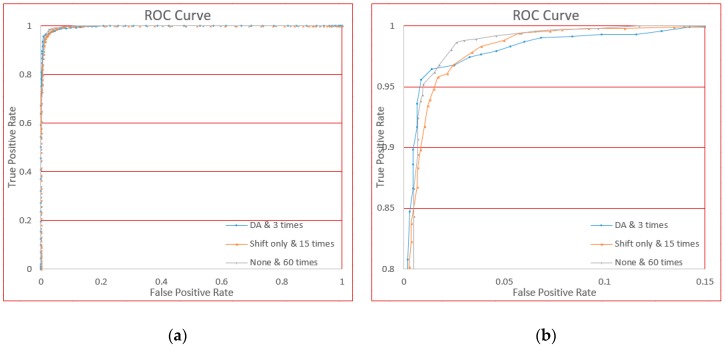
The ROC curve of using or not using data augmentation. (**a**) Is the global view and (**b**) is the local view for clarity.

To further study the influence of data augmentation, we also draw the ROC curve shown in Figure 19.

From Figure 19 we see the difference of using and not using data augmentation. The third curve (not using data augmentation) has slightly better performance, followed by the second curve (only using a shift operation), and the first curve (using full data augmentation) is still not far away from the others. This figure demonstrates that the effect of data augmentation on accuracy was negligible.

### 3.4. Analysis of Incremental Learning

Incremental learning is also based on user-friendly considerations. However, unlike data augmentation technology, incremental learning will increase system accuracy during use of the system. Using the system to achieve high reliability, we will compare the model after training with models that use incremental learning for a period of time.

To test this mechanism, we will test at 3 stages which is similar as for the “circle” gesture. The first stage is evaluated just after training. The second stage will use the model 5 times (which means 5 passes will contribute to incremental learning). The third stage is measured after 20 passes. The reason of choosing these 3 stages is we need to see the performance is gradually increasing. The first 5 uses have a more obvious effect than later uses. We will also make ROC curve for intuitive analysis.

Table 3 shows the FAR and FRR for different incremental learning times. The result after incremental learning is much better than before incremental learning.

**Figure 20:**
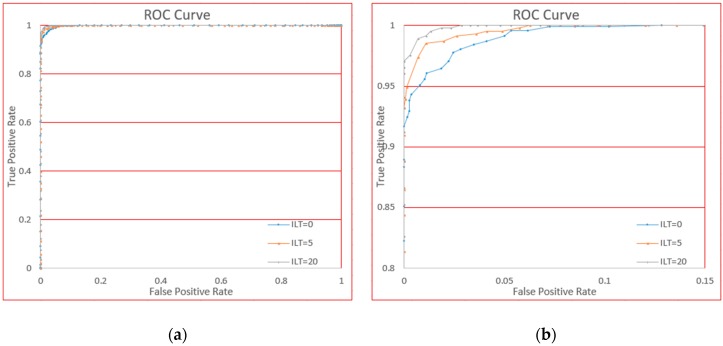
The ROC curve for different incremental learning times (ILT). (**a**) Is the global view and (**b**) is the local view for clarity.

The ROC curve is shown in Figure 20 for analysis.

From Figure 20 we see that the more the system is used, the higher the accuracy, which is consistent with the meaning of incremental learning. Using this system 20 times will result in almost zero authentication errors. From the curve, we could make a comparison how incremental learning will work: After training the system just could guarantee 1.24% FAR on 4.09% FRR according to our boundary making strategy, if we manually adjust the boundary we also could got approximately 3.5% EER (Equal Error Rate, when FAR is equal to FRR); 5 uses later, 1.10% FAR on 1.45% FRR will be achieved, EER is approximately 1.2% which is better than just after training; after 20 times using, this score will be 0.69% FAR on 1.08% FRR, approximately 0.9% EER will also be achieved. From this curve we could see our incremental learning mechanism worked over use.

### 3.5. Comparison of Previous Work

There have been significant research contributions in this field. Comparing the works in a very difficult thing because these works are contributive for this field but no obvious standard to evaluate an authentication is good or not, and every system has its unique design and methods, for example they have different user orientation, different training and testing methods, and different devices. Performance and false rate are very important in authentication system, but it depends on the how system works. For this, we select sensor, algorithm, false rate, and accuracy to make a comparison. 

We would like to briefly introduce their works. Zhao et al. [17] recorded the index finger track as information by Leap Motion, using HMM algorithm to make multi-class classification. In their experiment, they invited 4 users to test their system, these 4 users also attacked each other’s systems by imitating gestures, and in these processes FAR and FRR could be given. Aumi et al. [15] tried to divide gesture into two class: single finger and multiple fingers situation of total 15 users, in multiple fingers’ situation like us they got an average EER, then based on the EER they gave their system’s accuracy. Liu et al. [14] used accelerometer as their input device, and they employed 8 users and demonstrated that their system achieved 98.4% on same-day accuracy. Chen et al. [24] employed a multi-touch device as gesture input device, then by using SVM they could make this multi-class classification. They invited 32 volunteers, 22 of them are users and 10 of them are attackers, then they also gave the ROC curve like us, from that curve we could see the accuracy is over 98%. Yang et al. [25] invited 110 participants for performing the experiment, which is because their research is a comparison between text password and gesture password, then they got a result that using gesture password could get 98.90% at most. Chahar et al. [11] created spoofing samples by 3 individuals and then they got the result 1% FAR on 18.83% FRR to support their conditional mutual information maximization method. Sun et al. [26] have some similarity to our methods, they also made data smoothing and data normalization, but they used a DTW algorithm to make sequence alignment. Then they invited 19 volunteers using 3 smartphones to perform their experiment, and they got an average score of 0.27% FAR and 4.65% FRR, then they adjusted their threshold and got EER 1.86%.

We summarize these prior works and compare them to our work in Table 4.

From the table, we see that different studies use different evaluation standards, so it is very difficult to use a single standard for comparison. Considering the performance, our approach of zero incremental learning time (ILT) (i.e., just registered) has slightly better performance. However, after 20 passes of incremental learning, our approach achieves very low false rates among the studies.

### 3.6. Comparison of Other One-Class Classification Methods

In this section, we discuss some previous methods and compare their performance to our approach. From previous studies, we have observed several methods in one-class classification. We select one-class SVM (nearest neighbor), and the hidden Markov model for comparison.

#### 3.6.1. One-Class SVM

SVM plays a very important role in the development of machine learning, and one-class SVM performs well in one-class classification. Schölkopf et al. [27] proposed an approach using one-class SVM. Chen et al. [28] proposed using one-class SVM into image interval, which fits a tight hyper-sphere in the nonlinearly transformed feature space, and our experiment is based on this algorithm. They used a kernel-based SVM in content-based image retrieval, and the Gaussian kernel they used is of the following form:

(14) $$K\left(\left\|X-X_{C}\right\|\right)=e^{-\|X-X_{C}{\|}^{2}/2\sigma^{2}},$$

where X is any point in the space, $X_{C}$ is the kernel function center, and σ is the width parameter of the function. This kernel is a non-linear transformation, mapping the input space into a high-dimensional feature space, which gives the one-class SVM the ability to perform non-linear classification.

#### 3.6.2. Distance-Based K-Nearest Neighbor

K-nearest neighbor (KNN) is a classic algorithm in the machine-learning field. Usually it performs multi-class classification. Ramaswamy et al. [29] proposed an algorithm that mines outliers from large datasets. This algorithm ranks points based on the square of Euclidean distance between points. Because every classification of gesture authentication has only one label, the partition-based property will have little influence on the performance. The main concept of their algorithm is to evaluate the distance between the new incoming data and all other data. The distance value d is the maximum distance between a single point and all other points in the dataset, calculated as follows:

(15) $$d=\max\left({\mathrm{EuclDist}}^{2}\left(x,X\right)\right),$$

where x is one data point, and X is the set of all other points (excluding point x). Another parameter, k, is used to determine the number, k, of points within the distance, d, to a given new test point. By setting the k value, the classification can be made.

#### 3.6.3. Hidden Markov Model

The hidden Markov model (HMM) is a time-series probability model that describes the process of generating an unobservable random sequence from a hidden Markov chain, and generating an observation from each state to produce a random sequence of observations.

Zhao et al. [17] proposed a gesture authentication method based on HMM. This method is based on the flow of raw gesture data with some modification for fitting gesture format. First it calculates the state transition probability for each data sample time, then uses the training result to calculate the output probability of each state. 

#### 3.6.4. Comparison of Different Methods

We implemented the methods above and tested them using the same dataset. For testing their ability of one-class classification, we first used their default parameter values. Their FAR and FRR performance results appear in Table 5.

**Figure 21:**
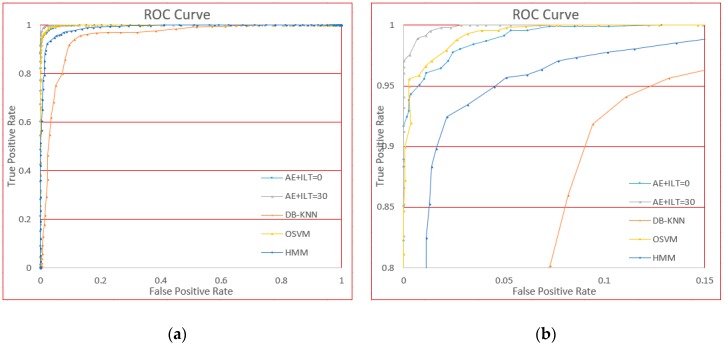
The ROC curves for different methods. (**a**) Is the global curve and (**b**) is the local curve for clarity.

The ROC curves based on adjusting the threshold parameter are shown in Figure 21.

From the figure, we can see that OSVM performs well compared to our methods (AE + ILT), because at stage 0 of incremental learning, the performance is approximately the same. However, DB-KNN and HMM are not very effective compared to the standard of authentication.

This comparison shows performance of our approach is relatively higher than the methods usually used in one-class classification.

## 4. Discussion

In this research, we proposed a system that used gesture movement as an authentication method, and employed many improvements to minimize user effort. The three stated requirements have been satisfied:

- Can authenticate from a single, customized gesture;
- Achieves high accuracy without an excessive number of gestures for training; and
- Continues learning the user’s gesture during use of the system.

The user could register his customized gesture by gesturing just three times, and it will still have high accuracy. Then the system will continuously learn the user’s gesture.

Recently, biometrics authentication has become a very popular form of authentication. Not only must the accuracy be satisfied but also the user-friendly consideration should also be addressed. Previous research has not considered these considerations. Our target system should satisfy these two requirements: Can authenticate by a single user’s customized gesture; achieves high accuracy without an excessive number of gestures for training. 

We used Leap Motion as the input device, which is an infrared-based depth camera. By continually reading the data from Leap Motion, we first filtered the data with median and Gaussian filters to remove some of the noise caused by device. Then we normalized the input gesture into a standard form for further processing. We mapped the 3D gesture information into three planes: X-Y, X-Z, and Y-Z. Then we used data augmentation to reduce the time for performing gestures. For one-class classification, we used an autoencoder to generate the output image from the input image, and compared their difference using the mean squared error function. Once the system is in use, if a user passes authentication, the new knowledge will be used for retraining as incremental learning. To evaluate the performance of our approach, an experiment was performed to evaluate the false rate of our system. After training, our system has 1.68% FAR and 3.58% FRR. After 20 passes, our system has 0.69% FAR and 1.05% FRR. This performance is acceptable for a behavior-biometrics authentication method [30].

### Future Work

As our system addresses user-friendliness, there are several application scenarios. We are exploring the possibility of this biometrics method replacing text passwords with a more user-friendly system. In the static physiological characteristic category, the fingerprint is a very successful authentication method. However, it still cannot ensure 100% accuracy. In some secure applications such as bank account applications, it needs more exploration. However, we believe that the potential of behavior characteristics will continually increase and eventually become a very popular and reliable authentication method.

## Figures and Tables

**Table 1:** False acceptance rate (FAR) and false rejection rate (FRR) score for different gestures after training and after 20 passes.

User ID	After Training		After 20 Passes	
	FAR	FRR	FAR	FRR
1	0.90%	4.70%	0.54%	0.74%
2	1.24%	4.09%	0.69%	1.08%
3	1.82%	3.46%	0.67%	1.18%
4	1.53%	3.66%	0.62%	1.02%
5	2.31%	2.73%	0.82%	1.06%
6	2.26%	2.82%	0.79%	1.22%
Avg.	1.68%	3.58%	0.69%	1.05%

**Table 2:** FAR and FRR score of using or not using data augmentation (DA).

Type	FAR	FRR
DA & 3 times	1.24%	4.09%
Shift Only & 15 times	1.67%	4.21%
None & 60 times	0.97%	4.80%

**Table 3:** FAR and FRR scores for different incremental learning times (ILT).

Type	FAR	FRR
ILT = 0	1.24%	4.09%
ILT = 5	1.10%	1.45%
ILT = 20	0.69%	1.08%

**Table 4:** Comparison of previous work to our approach.

Reference	Sensor	Algorithm	FAR	FRR	ERR ^1^	Accuracy
[17]	Leap Motion	HMM ^2^	1.65%	4.82%	-	95.21%
[15]	Intel Senze3D	DTW ^3^	-	-	2.9%	96.6%
[14]	Accelerometer	DTW	-	-	-	98.4%
[24]	Tablet	SVM ^4^	1.2%	2.6%	-	Over 98%
[25]	Tablet	ESM ^5^	-	-	-	98.90%
[11]	Leap Motion	CMIM ^6^	1%	18.83%	-	-
[26]	Smartphone	DTW	0.27%	4.65%	1.86%	-
Ours (ILT = 0)	Leap Motion	AE ^7^	1.68%	3.58%	-	-
Ours (ILT = 20)	Leap Motion	AE	0.69%	1.05%	-	-

^1^ Equal Error Rate. ^2^ Hidden Markov Model. ^3^ Dynamic Time Warping. ^4^ Support Vector Machine. ^5^ Experience Sampling Method. ^6^ Conditional Mutual Information Maximization. ^7^ Autoencoder.

**Table 5:** FAR and FRR for different methods.

Method	FAR	FRR
OSVM	1.37%	2.96%
DB-KNN	7.27%	19.80%
HMM	1.65%	4.82%
AE + ILT = 0	1.68%	3.58%
AE + ILT = 20	0.69%	1.05%

## Supplementary Materials

The following are available online at http://www.mdpi.com/1424-8220/18/10/3265/s1.
